# Factors associated with critical care needs in patients presenting with ST-elevation myocardial infarction: impact of early decompensation and culprit lesions

**DOI:** 10.3389/fcvm.2025.1625202

**Published:** 2025-10-21

**Authors:** Jack Jnani, Spencer F. Weintraub, Aditya Sood, Austin Cheng, Maikel Kamel, Riya George, Brandon Impastato, Shreya Srivastava, Ji-Cheng Hsieh, Yisrael Wallach, Allan Lin, Andrew Tsai, Jack Alboucai, Kishen Bulsara, Matthew Griffin, Miguel Alvarez Villela, Matthew Pierce

**Affiliations:** ^1^ Department of Medicine, Zucker School of Medicine, Northwell at North Shore University Hospital, Manhasset, NY, United States; ^2^ Department of Cardiology, Zucker School of Medicine, Northwell at North Shore University Hospital, Manhasset, NY, United States; ^3^ Department of Cardiology, Zucker School of Medicine, Northwell at Lenox Hill Hospital, New York, NY, United States

**Keywords:** ST-segment myocardial infarction (STEMI), critical care need, cardiac catheterization, culprit lesion, modified shock index, mortality

## Abstract

**Background:**

Patients with ST-elevation myocardial infarction (STEMI) are often admitted to the cardiac intensive care unit (CICU), though not all require advanced therapies. Identifying predictors of critical care need may improve triage and resource allocation.

**Methods:**

We performed a retrospective cohort study of 758 patients admitted with STEMI to a quaternary care CICU from 2018–2022. The primary outcome was critical care need, which was defined as use of mechanical ventilation, titratable infusions (vasoactive, sedative, or anti-arrhythmic), or mechanical circulatory support. Multivariable logistic regression was used to identify predictors of critical care need.

**Results:**

141 out of 758 patients (18.6%) utilized critical care resources, with the majority initiated before CICU admission (71%). We found that a history of chronic kidney disease (OR 4.3, 0.96–17.5, *p* = 0.05), STEMI in the post-COVID era (OR 2.7, 95% CI 1.45–5.09, *p* = 0.002), a Modified Shock Index on admission ≥ 0.93 (OR 4.04, 2.04–8.08, *p* < 0.001), and a lower ejection fraction (OR 0.97, 0.94–0.99, *p* = 0.007) were independent predictors of having critical care needs. Presence of a severe coronary stenosis (>70%), which was typically revascularized, did not increase critical care need, whereas multivessel coronary disease significantly did (OR 3.06, 1.64–5.83, *p* < 0.001).

**Conclusion:**

The majority of patients in our cohort did not require critical care resources after a STEMI, and a majority of those that did developed those needs prior to admission. A history of chronic kidney disease, elevated Modified Shock Index, reduced ejection fraction, and multivessel disease were associated with critical care needs while culprit vessel involvement was not.

## Introduction

Since the inception of cardiac care units (CCUs), admitting patients with ST elevation myocardial infarction (STEMI) to CCU and intensive care unit (ICU) settings has been the default (1–3). CCUs were lifesaving for these patients, significantly reducing mortality from STEMI complications (3, 4). Due to an evolution in revascularization technology, strategies, and abilities (5), STEMI mortality has decreased significantly to the point where some STEMI patients are considered low risk for major adverse cardiac events (MACE) and other traditional complications from STEMI (6, 7). However, the practice of admitting all STEMI patients to ICUs remains routine (1, 2), even for the growing number of these patients who are neither high risk nor critically ill (8).

As the acuity of the STEMI population has changed, so has the patient population in CCUs. Modern CCUs have transformed into Cardiac Intensive Care Units (CICUs) that now accommodate patients with higher acuity and increasing medical complexity, including sepsis, acute kidney injury, and respiratory failure (9, 10). As a result, there is a growing demand for ICU beds and critical care resources, making it imperative to reassess how these units are being utilized.

Given the improvement in STEMI outcomes, recent studies have focused on deescalating post STEMI care and have revealed over 82% of STEMI patients do not require critical care interventions (8), and many can be safely discharged within 48-hours post-revascularization (11). However, without a STEMI-specific risk stratification model, especially without an understanding of the timing of critical care needs and interventions and specific angiographic data in this population, ICU resources for these patients continue to be overused (8), driving up healthcare costs (12) without clear benefit to mortality or outcomes (13).

Our study seeks to identify timing of critical care needs and expand on clinical predictors of critical care needs in STEMI patients in order to improve triage decisions and ICU resource utilization in these patients.

## Materials and methods

### Study design

We retrospectively studied all patients (758) admitted with a diagnosis of a STEMI between 2018 and 2022 at Northwell Health's North Shore University Hospital, a quaternary academic medical center that serves the areas of Long Island, Queens, and surrounding boroughs of New York City. We selected the years of 2018–2022 to reflect modern STEMI management and capture the effects of the COVID-19 era on critical care needs. Our study was approved by the Institutional Review Board and informed consent was waived due to the retrospective nature of data collection.

### Inclusion and exclusion criteria

Patients were included if they had a confirmed diagnosis of STEMI and underwent primary percutaneous intervention (PCI), coronary artery bypass grafting (CABG), or received medical management alone. Only patients who had complete clinical and cardiac catheterization data, including baseline demographics, comorbidities, hemodynamic parameters, coronary angiograms, and procedural details, were included. Exclusion criteria included patients with non-STEMI or unstable angina, those transferred after primary-PCI occurred at another site, and patients with comfort care status on admission. While this was a single-center study, approximately 27% of patients were transferred from outside hospitals prior to PCI.

### Definition of critical care need

We defined “critical care need” based on institutional guidelines and prior literature (14–17). This included the use of mechanical or non-invasive ventilation, titratable vasoactive infusions, sedative infusions, and temporary mechanical circulatory support (tMCS). We utilized Pearson's Chi-Squared test to compare categorical data between groups, Fisher's exact test to compare categorical variables with less than 5 occurrences, and Welch's two-sample t test to compare continuous variables between the two groups. We also collected data on the initial setting of critical care need and its relationship to mortality.

### Data collection and management

Patient data were extracted from electronic medical records (EMR) and manually reviewed by investigators to ensure accuracy and capture highly granular clinical details often missing in automated data extraction. Study data were collected and managed securely using REDCap electronic data capture tools hosted at Northwell (18, 19). Given the manual nature of the chart review, we were able to minimize missing data and capture detailed clinical data.

### Statistical analysis

We performed a univariable logistic regression model to determine the association of each of our variables with critical care needs. The modified shock index, obtained from vital signs on admission, was calculated as the heart rate divided by the mean arterial pressure. We used a cutoff value of 0.93 for the Modified Shock Index based on prior literature showing that a Modified Shock Index ≥0.93 was associated with critical care need and mortality (14). We defined the COVID-19 pandemic era as occurring after 3/2020 given this was when the World Health Organization (WHO) classified it as a pandemic (20). Throughout the COVID era, including the first wave, our institution maintained a primary PCI strategy rather than thrombolysis, though system-level delays (e.g., personal protective equipment use, transfers, decreased staffing) were associated with delayed reperfusion despite unchanged catheterization rates (21).

Variables for regression analysis were selected based on prior literature (10–13) and clinical plausibility, focusing on factors associated with increased risk of mortality in STEMI patients. The logistic regression model was adjusted for collinearity and interaction terms were generated as needed and deemed non-significant. The multivariable regression model was valid after 10-fold cross validation to ensure a robust analysis.

Statistical analysis was performed using R-4.4.0 statistical computing software.

## Results

**Figure 1:**
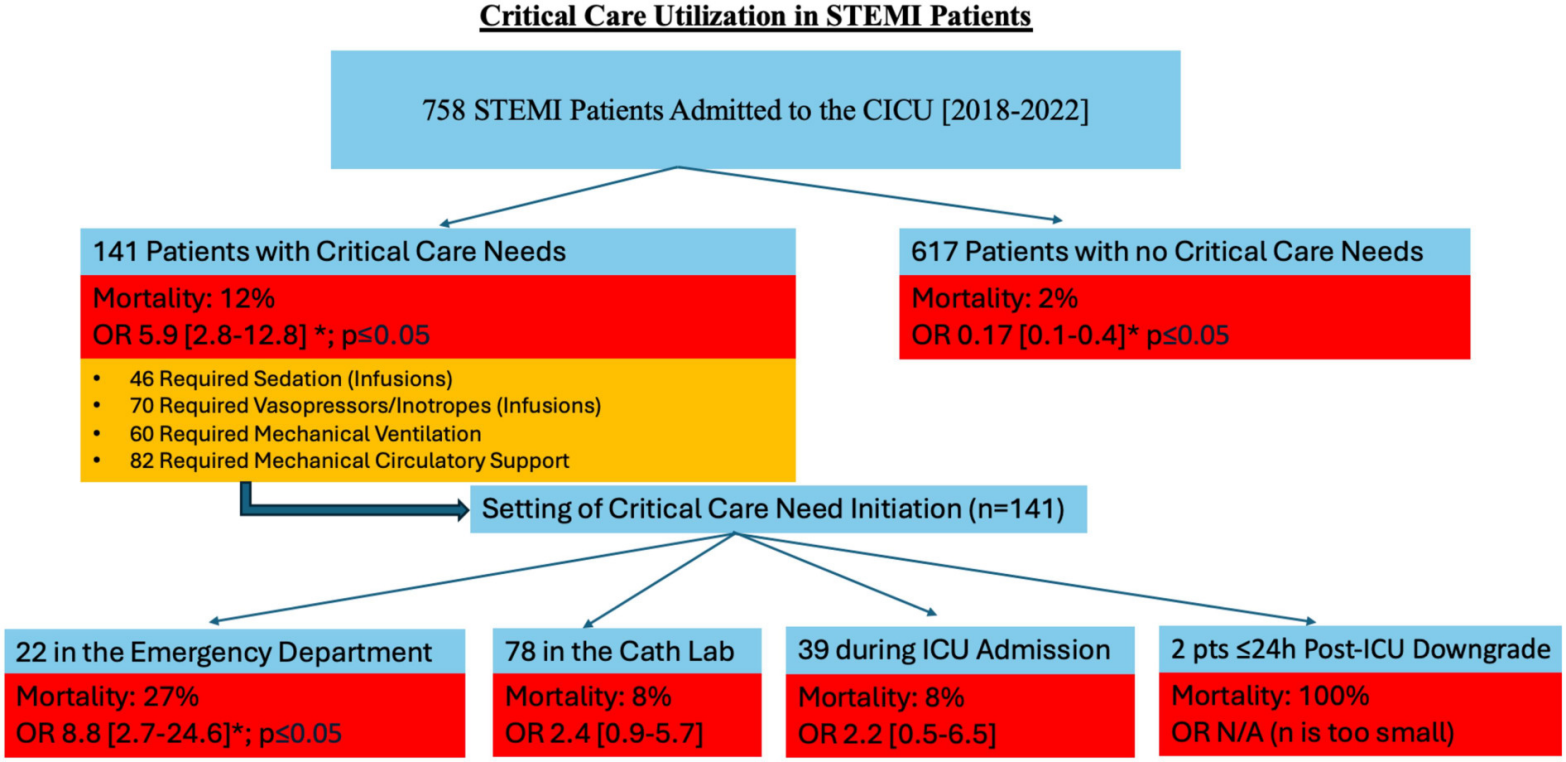
A Flowchart demonstrating critical care need in STEMI patients. Indicated in orange is the number of patients requiring specific critical care need, with some patients requiring multiple critical care therapies. An asterisk symbol represents statistical significance (*p ≤ 0.05). STEMI, ST-elevation myocardial infarction; CICU, coronary intensive care unit, OR, odds ratio; Cath, catheterization; ICU, intensive care unit; pts, patients; h, hours; N/A, not applicable, n, number of patients.

Among our study population, 141 out of 758 patients (18.6%) had a critical care need as described previously and 757 were admitted to a CICU (one died prior to admission). Of the 141 patients with critical care needs, most (71%) had critical care resources initiated prior to CICU admission: 22 (15.6%) in the ED, 78 (55.3%) in the cardiac catheterization lab, 39 (27.7%) in the CICU/other ICU, and 2 (1.4%) on the inpatient units within 24 h of ICU downgrade (Figure 1). In addition, those with a critical care need in the ED were more likely to die prior to discharge (OR 8.76, 2.7–24.6, *p* < 0.001), while those with a critical care need that started in the cardiac catheterization lab or CICU were not at significantly increased risk for mortality (Figure 1). Those without a critical care need (*n* = 617) had a significantly less chance of dying prior to discharge [OR 0.17 (0.1–0.4), *p* < 0.0001] (Figure 1).

**Figure 2:**
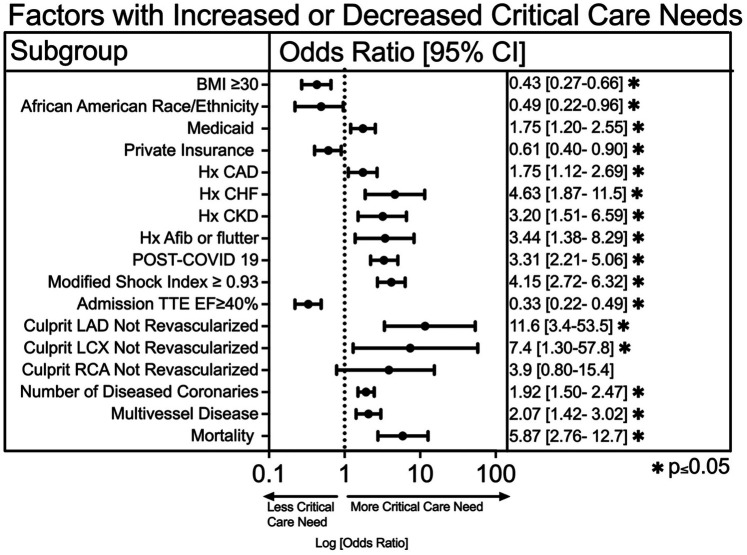
A univariable logistic regression analysis of demographic data, clinical characteristics, and cardiac catheterization data on critical care needs. An asterisk symbol represents statistical significance (*p ≤ 0.05).

We compared baseline demographics and clinical characteristics between the two cohorts (Table 1). A univariable logistic regression analysis was utilized to compare the effects of baseline clinical characteristics and revascularization on critical care needs. We found that patients with Medicaid insurance were significantly more likely (OR 1.75, 1.2–2.5, *p* = 0.004) to have a critical care need while those with private insurance were less likely (OR 0.61, 0.4–0.9. *p* = 0.013) to have a critical care need (Figure 2). Patients who were African American (OR 0.49, 0.22–0.96, *p* = 0.04) and those with elevated BMI ≥ 30 (OR 0.43, 0.27–0.66, *p* = 0.002) had reduced rates of critical care need. We also found that history of coronary artery disease (OR 1.8, 1.1–2.7, *p* = 0.014), congestive heart failure (OR 4.6, 1.9–11.5, *p* = 0.001), chronic kidney disease (OR 3.21, 1.5–6.6, *p* = 0.003) and atrial fibrillation or flutter (OR 3.4, 1.4–8.3, *p* = 0.009) were more common in the critical care needs group (Figure 2).

**Table 1:** Comparing Demographics and Baseline clinical Characteristics of Patients in the no critical care needs group (control group) vs. patients in the critical care needs group.

Characteristics	No critical care needs Group (Control Group)	Critical care needs Group	*p*-value^b^
	*N* = 617^a^	*N* = 141^a^	
Demographics			
Mean Age	61.46 (12.88)	62.42 (12.70)	0.4
Sex			
Female	161/617 (26%)	33/141 (23%)	0.5
Male	456/617 (74%)	108/141 (77%)	0.5
Race			
African American	75/617 (12%)	9/141 (6.4%)	0.05
Asian	111/617 (18%)	31/141 (22%)	0.3
Hispanic	24/617 (4%)	7/141 (5.0%)	0.6
Other	134/617 (22%)	39/141 (28%)	0.15
Caucasian	273/617 (44%)	55/141 (39%)	0.3
Insurance			
Medicaid	187/617 (30%)	61/141 (43%)	0.004
Medicare	184/617 (30%)	52/141 (37%)	0.11
Private	243/617 (39%)	40/141 (28%)	0.016
No Insurance	21/617 (3.4%)	4/141 (2.8%)	>0.9
Other Insurance	7/617 (1.1%)	3/141 (2.1%)	0.4
Multiple Insurance	66/617 (11%)	15/141 (11%)	>0.9
Mean BMI	28.71 (5.45)	27.25 (4.83)	0.002
Medical/Social History			
History CAD	101/617 (16%)	36/141 (26%)	0.015
Hx of CHF	10/617 (1.6%)	10/141 (7.1%)	0.001
Hx of CKD	19/617 (3.1%)	13/141 (9.2%)	0.004
Hx of COPD	20/617 (3.2%)	5/141 (3.5%)	0.8
Hx of Diabetes Mellitus (Type 1 or 2)	193/617 (31%)	55/141 (39%)	0.09
Hx of Hypertension	349/617 (57%)	88/141 (62%)	0.2
Hx of Hyperlipidemia	273/617 (44%)	64/141 (45%)	0.9
Hx of Atrial Fibrillation/Flutter	12/617 (1.9%)	9/141 (6.4%)	0.008
Current Alcohol Abuse	29/617 (4.7%)	7/141 (5.0%)	0.8
Current Smoker	145/587 (25%)	30/131 (23%)	0.7
Cardiac Catheterization Data			
Culprit Coronary Artery			
Left Main (>70% Occlusion)	3/616 (0.5%)	3/136 (2.2%)	0.08
LAD	255/616 (41%)	74/136 (54%)	0.007
RCA	245/616 (40%)	34/136 (25%)	0.001
LCx	50/615 (8.1%)	17/136 (13%)	0.13
Number of Diseased Coronaries^c^			
0	35/616 (5.7%)	6/136 (4.4%)	0.7
1	392/616 (64%)	65/136 (48%)	<0.001
2	160/616 (26%)	39/136 (29%)	0.59
3	29/616 (4.7%)	22/136 (16%)	<0.001
4	0/616 (0%)	4/136 (2.9%)	0.001
Late-presentation STEMI	38/592 (6.4%)	13/124 (10%)	0.12
Other Characteristics			
COVID-19 Pandemic Era (after 3/2020)			
Yes	295/617 (48%)	106/141 (75%)	<0.001
No	322/617 (52%)	35/141 (25%)	<0.001
Baseline EF	47.76 (12.76)	40.09 (14.10)	<0.001
Modified Shock Index ≥ 0.93	93/563 (17%)	55/122 (45%)	<0.001
Mortality	13/581 (2.2%)	16/135 (12%)	<0.001

Bolded values indicate statistical significance (*p* ≤ 0.05).

BMI, body mass index; Hx, history of; TTE, transthoracic echocardiography; EF, ejection fraction; CAD, coronary artery disease; CHF, congestive heart failure; CKD, chronic kidney disease; COPD, chronic obstructive pulmonary disease; LAD, left anterior descending coronary artery; RCA, right coronary artery; LCx, left circumflex coronary artery.

^a^ n/N (%); Mean (SD).

^b^ Welch's Two Sample *t*-test; Pearson's Chi-Squared Test; Fisher's exact test.

^c^ Diseased Coronaries included critical lesions involving the Left Main, Left Anterior Descending coronary, Right Coronary, and Left Circumflex Coronary arteries.

The majority of critical (>70%) stenoses in culprit coronary arteries were revascularized, including 100% of Left Main (LM), 96.4% of Left Anterior Descending (LAD), 91.3% of Left Circumflex (LCX), and 96.8% of Right Coronary Artery (RCA). However, in the small subset of unrevascularized culprit vessels, the need for critical care was significantly higher. Specifically, a critical stenosis in an unrevascularized LAD had significantly more critical care need (OR 11.6, 95% CI 3.4–53.5, *p* = 0.0003), as was an unrevascularized LCX (OR 7.4, 95% CI 1.3–57.7, *p* = 0.03). Multivessel disease had significantly increased critical care need (OR 2.07, 95% CI 1.4–3.0, *p* < 0.001), as was an increasing number of critically diseased coronary vessels (OR 1.9, 95% CI 1.5–2.5, *p* < 0.001) (Figure 2). Lastly, patients requiring critical care had significantly higher mortality compared to those who did not (OR 5.9, 95% CI 2.8–12.8, *p* < 0.0001) (Figure 2).

**Figure 3:**
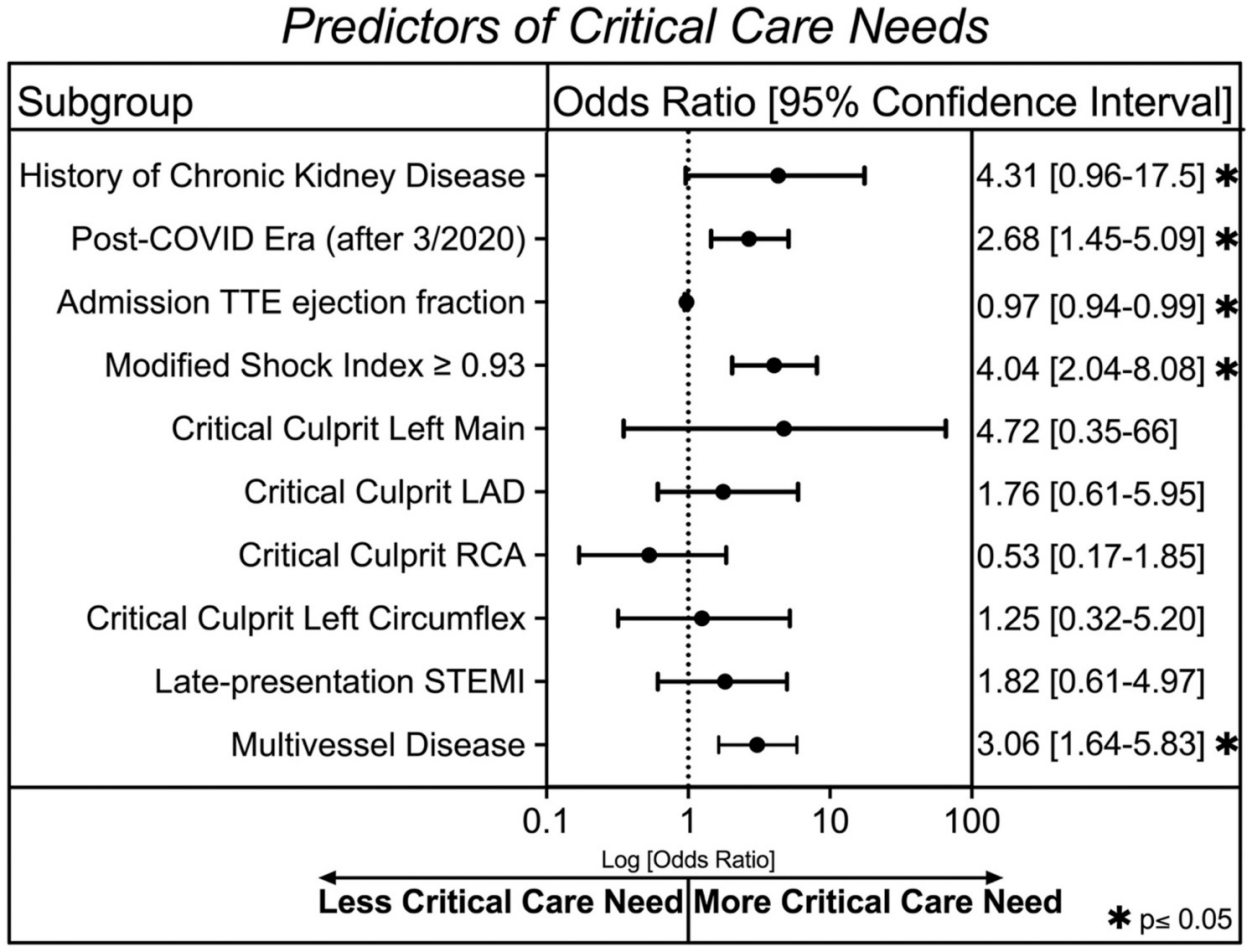
A multivariable logistic regression analysis after 10-fold cross validation showing clinical characteristics associated with critical care need in our cohort. An asterisk symbol represents statistical significance (*p ≤ 0.05).

We also utilized a multivariable logistic regression statistical analysis to compare variables across groups with and without a critical care need (Figure 3). Factors associated with critical care need included history of chronic kidney disease (OR 4.3, 95% CI 0.96–17.5, *p* = 0.045), having a STEMI in the COVID era (OR 2.7, 95% CI 1.5–5.1, *p* = 0.002), and a Modified Shock Index (MSI) ≥ 0.93 on admission (OR 4.04, 95% CI 2.04–8.08, *p* < 0.001). Higher ejection fraction was protective (OR 0.97 per 1% increase, 95% CI 0.94–0.99, *p* = 0.007). Catheterization data showed that a critical stenosis (>70%) in a proximal culprit artery was not associated with critical care need, possibly because these lesions were routinely revascularized. In contrast, multivessel disease was significantly associated with critical care need (OR 3.06, 95% CI 1.6–5.8, *p* < 0.001).

## Discussion

The major findings of this study are fourfold. One, despite the entire STEMI cohort being admitted to the ICU as part of our hospital's protocol, only 18.6% of these patients were critically ill. Second, the majority of these ICU needs arose before ICU admission. Three, STEMI patients with non-revascularized proximal LAD or LCx lesions and multivessel lesions had increased burden of critical care needs, while successful PCI of culprit lesions mitigated ICU needs regardless of the culprit vessel. Finally, our study also found that chronic kidney disease and low ejection fraction were independently associated with critical care needs in this cohort.

Previous studies have assessed the general risk of mortality in STEMI patients (15–17) and have noted the over-triage of these patients to intensive care settings in the absence of critical illness (22, 23). Our study validates this data but also provides novel insights not previously reported in this setting by examining granular data that is used in real world settings but not available in larger registries, such as the timing of critical care needs for this cohort and also culprit lesion involvement. We found that less than twenty percent of our STEMI patients had critical care needs. This percentage was consistent with larger, multi-centered and registry-based studies (22, 24). However, our study differs and is unique from these larger studies in that it specifies that most critical care needs in this cohort occur before ICU admission, occurring mainly in the catheterization lab. Notably, patients who required critical care interventions in the ED were significantly more likely to die prior to discharge, which likely reflects those presenting in extremis or with very high initial acuity. This implies that the majority of patients who do not require critical care interventions prior to or during revascularization for a STEMI are unlikely to develop a need for them afterwards. Therefore, STEMI patients who have had an uncomplicated hospital course up until the time of admission have a low risk of adverse events if admitted to a non-ICU setting or if discharged from the hospital within 24–48 h. These findings help build on the current literature by giving more granular detail to the overall rate of critical illness in STEMI patients, which were generally cited by larger, registry-based studies. They also challenge the current standard of care and guideline recommendation (25) where the majority of STEMI patients are admitted to the CICU even in the absence of a critical care need (1–3). These results further support the growing paradigm that low-risk STEMI patients post revascularization can be triaged not only to telemetry floors (26) but even potentially discharged from the hospital (17).

We are also the first to identify the impact of specific coronary anatomy on critical care needs in STEMI patients. We demonstrated that patients with non-revascularized proximal LAD or LCX lesions and multivessel lesions are associated with an increased burden of critical care needs. Conversely, successful PCI of culprit lesions mitigated ICU needs regardless of the culprit vessel. This is notable in that many current risk stratification scores designed to identify STEMI patients at low risk for complications and MACE that may be eligible for early hospital discharge designate anterior wall infarct, even if revascularized, as contributing to a higher risk profile (6, 7, 27). Other studies have also cited the perceived increased risk in LAD revascularization, especially its proximal segment, perhaps due in part to the amount of myocardium it supplies and the fact that until recently, surgical intervention was the preferred method of revascularization (28–30). Our study did find that kidney disease and low ejection fraction were associated with critical care needs, consistent with other risk stratification studies (31, 32) that found these variables conferred higher risk in STEMI patients.

However, the risk factors for critical illness in STEMI patients identified in this study were not always consistent with all risk factors cited in the current literature for this population. This can be explained by the fact that these studies have not specifically examined the characteristics that carry higher or lower risk for critical care needs in STEMI patients. Current risk models, including PAMI-II, APACHE III, and Modified Zwolle risk scores focus primarily on mortality and complication risk to help determine suitability for early hospital discharge rather than critical care needs (15–17). These tools vary in their approach and may not fully address the complexities of patient needs in the acute setting or predict critical care requirements accurately. For example, while the Modified Shock Index was a strong predictor of critical care needs in our study and in prior literature (14), it lacks integration of critical angiographic and clinical parameters, limiting its applicability as a stand-alone triage model. Our findings suggest that incorporating clinical factors, hemodynamic data, and angiographic specifics into a risk score could significantly improve risk stratification and ICU resource allocation for STEMI patients. As multiple studies have shown that CICU stays and prolonged inpatient admissions for low-risk STEMI patients do not improve clinical outcomes and are not associated with improved patient satisfaction (33, 34) but do contribute to rising hospitalization costs (8, 12, 13), finding an accurate method to determine which cohort of STEMI patients truly need ICU admission is essential.

All patients in our cohort were admitted to the CICU regardless of insurance status, as per our institutional STEMI protocol. Therefore, these differences may reflect underlying socioeconomic and comorbidity differences, not differences in ICU access. The association of Medicaid insurance with greater critical care needs likely reflects underlying socioeconomic disparities, comorbidity burden, and barriers to longitudinal care. These findings underscore the need for health system and policy-level strategies to address disparities in outcomes for STEMI patients.

This study has several limitations. It is a retrospective, single-center design, which may limit generalizability and introduce confounding, and therefore should be considered hypothesis-generating. We defined the post-COVID era as STEMI admissions after March 2020 without confirming individual infection status; thus, higher critical care use may reflect pandemic-related factors rather than COVID illness itself. The generalizability of this variable to non-pandemic contexts is limited. We did not have precise door-to-balloon times for transferred patients, which restricted assessment of treatment delays. However, the higher critical care needs in unrevascularized LAD and LCx lesions emphasize the importance of timely and complete reperfusion. Culprit vessel location was not predictive once successful PCI occurred. Although adequately powered for the primary analysis, the study may have been underpowered to detect associations in certain subgroups, such as non-revascularized patients. We defined “critical care need” by resource utilization, which overlaps with therapies used in cardiogenic shock, suggesting that our findings may have relevance for early triage in this high-risk population. Finally, only a small number of patients underwent CABG evaluation, and while these cases typically require critical care, their low frequency meant they likely had little influence on the overall findings.

Future directions include expanding this work to a multicenter registry or national database, which would provide greater statistical power, enhance external validity, and allow for cost-effectiveness analyses. This represents a logical next step that we are actively exploring within our network.

## Conclusions

Most STEMI patients requiring critical care had already decompensated before CICU admission, and clinical factors such as chronic kidney disease, elevated Modified Shock Index, and multivessel lesions helped identify those at higher risk. Our findings challenge routine ICU admission for all STEMI patients and highlight the need for refined risk stratification to optimize resource utilization and improve patient outcomes.

## Data Availability

The original contributions presented in the study are included in the article/Supplementary Material, further inquiries can be directed to the corresponding author.
